# Autoimmune Hemolytic Anemia Induced by Levofloxacin

**DOI:** 10.1155/2014/201015

**Published:** 2014-06-12

**Authors:** Marwan Sheikh-Taha, Pascale Frenn

**Affiliations:** Lebanese American University, P.O. Box 36, Byblos, Lebanon

## Abstract

Drug-induced autoimmune hemolytic anemia is a rare condition. We report the case of a 32-year-old white female who presented to the emergency department with generalized fatigue, fever, and jaundice. The patient reported using levofloxacin few days prior to presentation for urinary tract infection. The patient had evidence of hemolytic anemia with a hemoglobin of 6.7 g/dL which dropped to 5 g/dL on day 2, the direct Coombs test was positive, indirect bilirubin was 5.5 mg/dL, and LDH was 1283 IU/L. Further testing ruled out autoimmune disease, lymphoma, and leukemia as etiologies for the patient's hemolytic anemia. Levofloxacin was immediately stopped with a gradual hematologic recovery within few days.

## 1. Case Report

Fluoroquinolones have become an increasingly popular class of antibiotics for use in a variety of infections. Although they are very useful agents, they are associated with a number of adverse events and some with considerable clinical significance. Levofloxacin is a third-generation fluoroquinolone with a broad spectrum of activity against Gram-positive and Gram-negative bacteria and atypical respiratory pathogens. Adverse events commonly associated with levofloxacin include gastrointestinal and CNS toxicity (mainly headache and dizziness), as well as other adverse events including EKG abnormalities, disrupted glucose metabolism, phototoxicity, hypersensitivity and skin disorders, tendon and joint disorders, and liver toxicity [1].

A 32-year-old white female, with an unremarkable past medical history, presented to the emergency department with generalized fatigue, fever, and decreased oral intake. The patient reported jaundice, nausea, and vomiting for few days as well. The only medication that she was taking was levofloxacin at a dose of 250 mg orally once daily for urinary tract infection that was diagnosed one week prior to presentation. She had no history of blood transfusions, drug abuse, jaundice, or anemia. On admission, the patient was in distress complaining mainly of fatigue and her vital signs included temperature, 38.3°C; blood pressure, 100/60 mm Hg; and heart rate, 107 beats per minute. The patient reported no allergies to drugs or food.

General physical exam was unremarkable except for icteric sclera and yellowish discoloration of the skin. The hemoglobin was 6.7 g/dL and dropped to 5 g/dL on day two, and marked spherocytosis was noted on peripheral smear while red blood cell indices were normal. The direct Coombs test was positive, indirect bilirubin was 5.5 mg/dL, and LDH was 1283 IU/L. Further testing ruled out autoimmune disease, lymphoma, and leukemia. Liver and renal function tests were within normal.

The patient was diagnosed with drug-induced autoimmune hemolytic anemia (DIIHA) secondary to fluoroquinolone use and was treated supportively with intravenous hydration and red blood cells transfusions. Levofloxacin was discontinued and was replaced by ceftriaxone. The patient also received intravenous methylprednisolone and acetaminophen. She responded well to the above-mentioned measures and had uneventful recovery and was discharged after 6 days of hospitalization. Repeated serial hematologic laboratory studies showed a steady rise in hemoglobin and reversal of her anemia.

Autoimmune hemolytic anemia is an immune disorder caused by antibodies directed against autologous red cells. DIIHA is rare and is roughly estimated to be around 1 in 1 million of the population [2]. The three groups of drugs that are most commonly implicated in causing DIIHA include antimicrobials (mainly cefotetan, ceftriaxone, and piperacillin), anti-inflammatory, and antineoplastics [3]. A PubMed search revealed one case report of levofloxacin-induced autoimmune hemolytic anemia [4] while there are several published case reports of hemolytic anemia with other fluoroquinolones including ciprofloxacin. Temafloxacin was withdrawn from the market in 1992, four months after approval in USA, due to this adverse effect (95 cases) [5].

Drug-induced immune hemolysis is classified according to three mechanisms of action. The first mechanism is drug absorption, also known as hapten induced in which a medication attaches to the red blood membrane and stimulates IgG antibody production. When the antibody binds the cell membrane, it causes extravascular hemolysis. The second mechanism is the immune complex mechanism, in which the drug induces IgM antibody production. When the drug-antibody complex binds to the red blood cell membrane, it initiates complement activation, leading to intravascular hemolysis. The third mechanism is the autoantibody where the drug induces the production of antierythrocyte IgG antibodies via unknown mechanisms and causes an extravascular hemolysis [6]. The exact mechanism by which levofloxacin induces DIIHA remains to be determined. It should be noted that quinine and quinidine have been implicated in severe cases of hemolytic anemia and their chemical structure is similar to fluoroquinolones [4]. The main treatment for DIIHA is to stop the offending drug and blood transfusion if the patient is sufficiently anemic [7]. In addition, steroids may also be needed [8].

This patient's adverse reaction can be attributed to levofloxacin because of the temporal relationship between use of the drug and the development of the illness. In addition, the patient had no risk factors for developing hemolytic anemia, and no underlying etiology was evident. In addition, the patient's presentation was typical of that of “temafloxacin syndrome”: fever, chills, and jaundices, a mean of 6.4 days after starting therapy [5]. On the other hand, our patient did not have renal sequelae while 57% of patients with temafloxacin syndrome did. Use of the Naranjo et al. [9] adverse-reaction probability scale revealed a probable association between the DIIHA and levofloxacin (score of 6).

Health care providers should be aware of the serious and life threatening DIIHA that may occur with levofloxacin use. Timely recognition of the drug causing DIIHA should not be underestimated as failure to do so can result in continuation of the offending agent and worsening of the patient's hemolytic anemia.
